# MicroRNA-127 inhibits cell proliferation via targeting Kif3b in pancreatic β cells

**DOI:** 10.18632/aging.101835

**Published:** 2019-03-01

**Authors:** Ziyang Shen, Hemin Jiang, Hsiang-Ting Hsu, Li Qian, Qi Fu, Min Shen, Shu Chen, Tao Yang

**Affiliations:** 1 Department of Endocrinology and Metabolism, The First Affiliated Hospital of Nanjing Medical University, Nanjing 210029, China; * Equal contribution

**Keywords:** β cells, miR-127, cell viability, insulin secretion, EVs

## Abstract

MicroRNAs (miRNAs) have been implicated in β cells dysfunction. Previous studies indicated that miR-127 was specifically abundant in β cells and one of its target genes, Kif3b, promoted cell proliferation. However, the impact of the miR-127-Kif3b axis on β cells remains unknown. In this study, we revealed that miR-127 level was declined both in islets from the mice with a high-fat diet and in MIN6 cells with elevated glucose treatment. The elevated level of miR-127 attenuated β cell proliferation by repressing Kif3b expression without affecting apoptosis and cell cycle, and it dampened insulin secretion. Moreover, β cell-derived miR-127 could also affect the islet endothelial cell-line, MS1, *in vitro* via the transfer of extracellular vesicles (EVs). Treating MS1 cells with the EVs secreted by MIN6 cells exhibited a higher ability in cell migration and tube formation. However, this effect was abolished by the miR-127 inhibitor co-cultured with EVs-treated MS1 cells. Thus, we define that miR-127 is a crucial regulator of insulin secretion and cell proliferation in pancreatic β cells as well as a potential functional regulation factor in islet endothelial cells.

## Introduction

Diabetes mellitus is characterized by elevated blood glucose and it affects more than 400 million people worldwide; therefore, it has been recognized as one of the global public health issues [1]. Beta cell dysfunction and loss of β cell identity lead to insufficient insulin secretion and contribute to the pathogenesis of type 2 diabetes (T2D) [2]. Previous studies demonstrated that the aberrant expression of certain transcription factors was related to β cell dysfunction [3]. However, to explore exact mechanisms underlying β cell dysfunction is still in need of further investigation. Emerging studies have highlighted the key roles of miRNA in β cell function including apoptosis, insulin secretion, etc [4].

MiRNA is a class of endogenous 18-22 nucleotides non-coding RNAs that bind to the 3’ untranslated region of target genes to induce mRNA deregulation and translational repression [5]. Accumulating evidence shows that miRNAs are involved in various biological processes including apoptosis, differentiation, proliferation, carcinogenesis and cell metabolism [6,7]. Moreover, several miRNAs play an important role in mouse pancreas development, insulin gene expression and insulin secretion [8]. For example, it was demonstrated that miR-375 and miR-15a positively regulated insulin secretion by targeting MTPN and UCP-2, respectively [9,10], whereas miR-133a acted as a negative regulator in insulin production [11].

Both of miR-127-3p and miR-127-5p are processed from pre-miR-127; hereafter, miR-127-3p, the predominant form, is referred to as miR-127. MiR-127 is expressed in murine embryos and plays a role in the lung development [12]. It has also been identified as a crucial modulator in cancer cell proliferation, differentiation and migration, either as a tumor suppressor or an oncogenic factor dependent on the tumor type [13,14]. In the context of islets, it has been also reported that miR-127 was strongly expressed in human pancreatic islets and specifically enriched in β cells [15]. Similarly, it was demonstrated that miR-127 was highly expressed in murine islets next to miR-375, which was ranking at the first place of expression level in murine islets and was well studied in the field of islet research [16,17]. However, little evidence has revealed the function of miR-127 in islets, especially in β cells, and the studies were limited to a correlation between its expression level in pancreatic islets and insulin secretion [18]. Moreover, Donato *et al.* have demonstrated that levels of miR-127 in extracellular vesicles (EVs) from T2D patients’ plasma were significantly elevated in comparison with those from healthy control subjects [19]. Accumulating evidence suggested that EVs were involved in the cross-talk between donor cells and nearby recipient cells [20]. We hypothesized that miR-127 might regulate β cell viability and function by promoting or repressing its target genes as well as affect nearby tissues via EVs transfer. Here, we reveal that miR-127 down-regulates β cell proliferation and insulin secretion. It can furthermore promote vessel formation of islet endothelial cells via EVs transfer *in vitro*.

## RESULTS

### miR-127 is highly expressed in mouse islets

**Figure 1:**
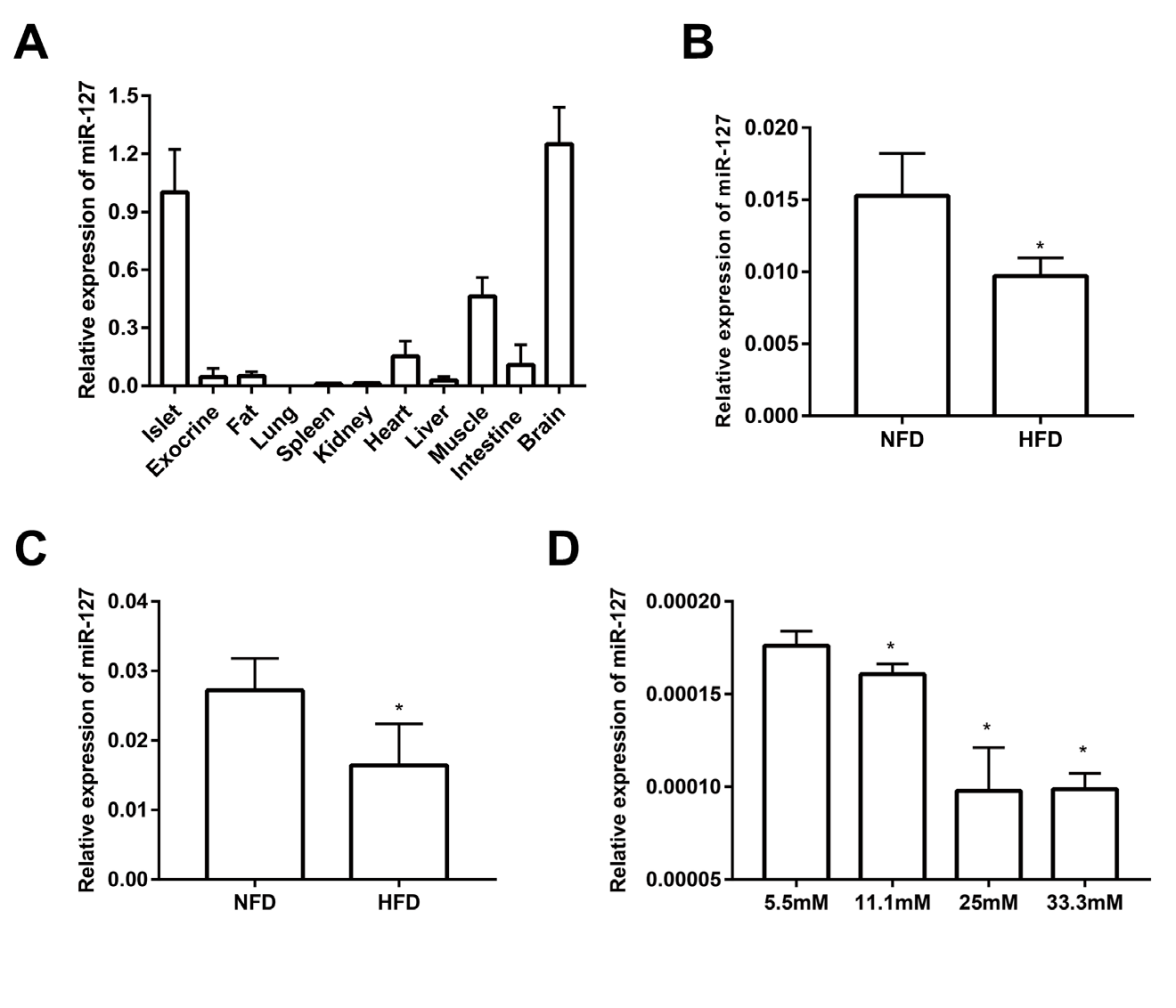
**miR-127 was dominantly expressed in mouse islets.** (**A**) QRT-PCR analysis of relative miR-127 expression in various isolated tissues from 8-week-old C57BL/6 mice (n=4). The level of miR-127 in mouse islets were measured in 5-week (**B**) and 20-week (**C**) old HFD mice and age-matched NFD mice (n=4) by qRT-PCR. NFD, normal fat diet; HFD, high fat diet. (**D**) MIN6 cells were cultured with the indicated concentrations of glucose (5.5, 11.1, 25, or 33.3mM) for 24 h. Subsequently, miR-127 expression was measured by qRT-PCR. U6 was used as the internal control. The values are presented as the means ± SD. *p<0.05.

As shown in Figure 1A and it is consistent with previously published data [21], miR-127 is predominantly expressed in the islets and brain when compared with other tissues including fat, kidney, heart, liver, spleen, lung, muscle and intestine. Moreover, miR-127 is specifically enriched in islets compared with pancreatic exocrine tissue. To investigate the role of miR-127 in the pathogenesis of T2D, we examined its level in the islets from HFD mice. The results showed that the expression of miR-127 was significantly downregulated with HFD for 1 week (Figure 1B) or 16 weeks (Figure 1C). To determine whether miR-127 expression was regulated by glucose, MIN6 cells were exposed to different concentration of glucose from 5.5mM to 33.3mM for 24 h. We observed that the expression level of miR-127 was diminished along with the increase of glucose concentration (Figure 1D). Taken these data together, it miR-127 might be involved in β cell function.

### MiR-127 inhibits β cell proliferation and insulin secretion

**Figure 2:**
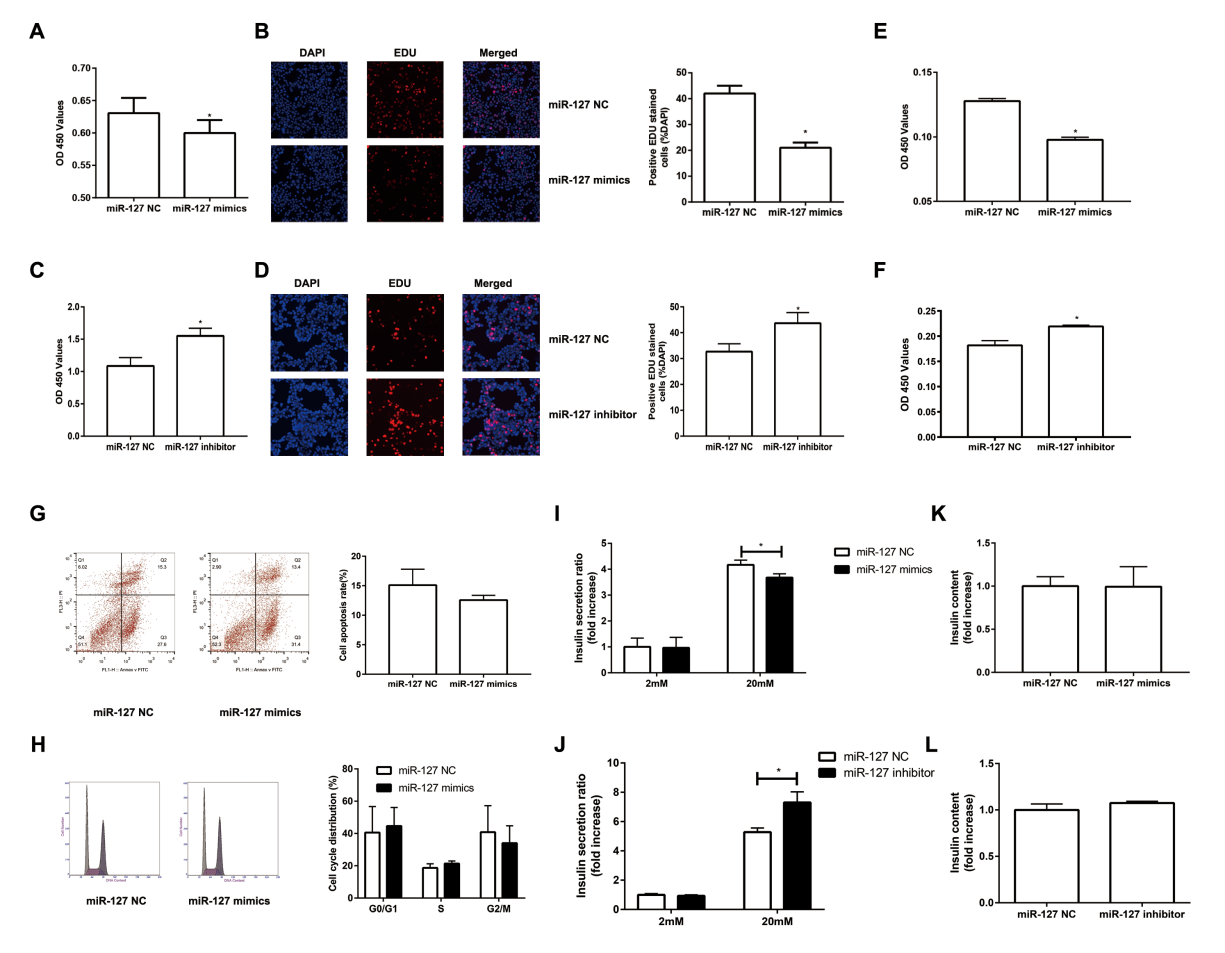
**Overexpression of miR-127 attenuated cell proliferation and insulin secretion.** MIN6 cells and primary islet cells were transfected with miR-127 mimics, miR-127 inhibitor or matched control. Forty-eight hours after transfection with miR-127 mimics, MIN6 cell viability was assayed by the CCK-8 assay (**A**) and EDU assay (**B**). CCK-8 assay (**C**) and EDU assay (**D**) were used to determine cell viability in MIN6 cells after transfection with miR-127 inhibitor. Primary islet cells proliferation was measured after transfection with miR-127 mimics (**E**) and miR-127 inhibitor (**F**) using CCK-8 assay. (**G**) Apoptosis was evaluated by flow cytometry with Annexin V-FITC and PI staining, and the quantitative analysis of apoptotic cell frequency was shown. (**H**) MIN6 cells were harvested and cycle distribution monitored with flow cytometry, and a quantitative analysis of the cell cycle distribution was shown. Relative level of insulin secreted in the supernatant (**I**, **J**) and cellular insulin content (**K, L**) were measured by ELISA and normalized by total protein content. The level of insulin in miR-127 NC group was defined as 1 and the results were shown as a relative fold of increase. The values are presented as the means ± SD. *p<0.05.

To investigate the impact of miR-127 on cell proliferation, we augmented or reduced the level of miR-127 by transfecting MIN6 cells with miR-127 mimics or miR-127 inhibitor, respectively, followed by CCK-8 assay and EDU assay to detect cell proliferation. We found that the proliferation was repressed in the cells with miR-127 mimics transfection (Figure 2A, B). Cell proliferation was promoted in MIN6 cells transfected with miR-127 inhibitor (Figure 2C, D). Similar results were confirmed in isolated islet cells (Figure 2E, F). Then we examined whether miR-127 mimics induced apoptosis or affected cell cycle in MIN6 cells. No significant changes were observed in apoptosis and cell cycle between miR-127 NC and miR-127 mimics transfected cells (Figure 2G, H). To examine the effect of miR-127 on insulin synthesis and secretion, the content of secreted insulin in the culture medium and the intracellular content of insulin in cells transfected with different miR-127 molecules were measured by ELISA. Insulin secretion in response to glucose stimulus was repressed in the miR-127 mimics group (Figure 2I) while it was promoted in the miR-127 inhibitor group (Figure 2J). However, enhancing or reducing the level of miR-127 did not influence intracellular content of insulin in MIN6 cells (Figure 2K, L). Collectively, these results demonstrated that miR-127 functionally suppressed cell proliferation and insulin secretion.

### MiR-127 directly binds to the Kif3b 3′-UTR

**Figure 3:**
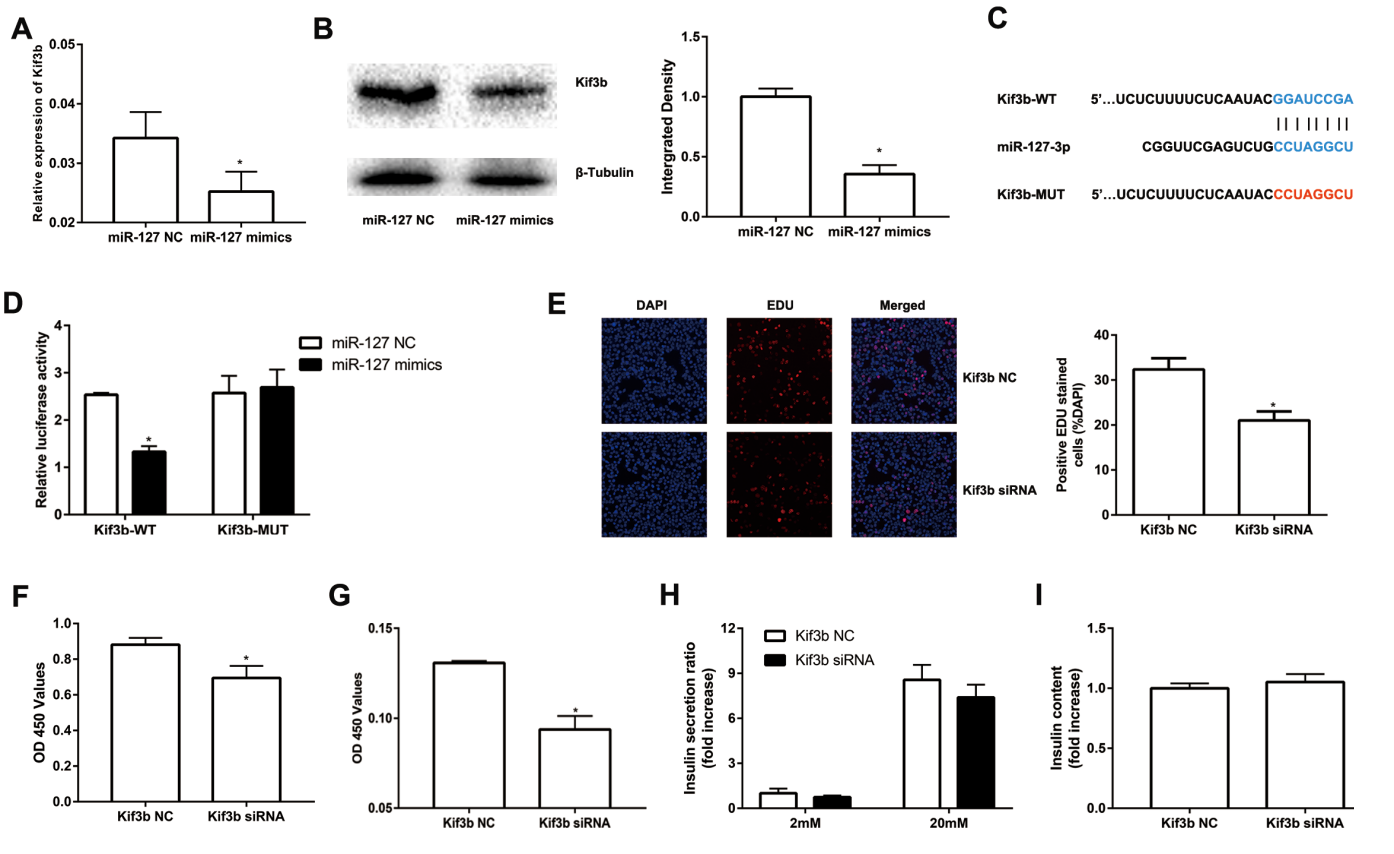
**miR-127 suppressed cell proliferation by targeting Kif3b.** Effects of overexpressed miR-127 on Kif3b mRNA (**A**) and protein expression (**B**) were estimated by qRT-PCR and Western blot respectively. (**C**) Schematic representation shows the predicted target sites of miR-127 at the 3′-UTR of Kif3b. A mutation in the seed matches is refereed as Kif3b-MUT. (**D**) Luciferase assay of the reporter plasmids harboring the intact or mutant Kif3b-3′UTR in MIN6 cells. EDU assay (**E**) and CCK-8 assay (**F**) were used to evaluate MIN6 cell proliferation. (**G**) CCK-8 assay was applied to evaluate primary islet cells viability. Insulin secreted in the supernatant (**H**) and cellular insulin content (**I**) were measured by ELISA and normalized to total protein content. The level of insulin in Kif3b NC group was defined as 1 and the results were shown as a relative fold of increase. The values are presented as the means ± SD. *p<0.05.

To further evaluate the molecular mechanism of miR-127, we screened potential target genes of miR-127 with the algorithm tools, TargetScan and miRDB. Four target genes including Kif3b, Fjx1, Sept7 and Wnt7a were predicted as the target genes of miR-127 with both of the algorithm tools. However, only the expression of Kif3b was suppressed, at the level of either mRNA or protein, in miR-127 mimics transfected cells (Figure 3A, B; Supplementary Figure 1A-C). Moreover, the expression of Kif3b was upregulated in islets of HFD mice in comparison with those from NFD mice and it was also enhanced in elevated glucose (Supplementary Figure 1D, E). Kif3b, which has been reported as a proliferation-regulatory gene was thus selected for further study [22]. To confirm the binding relationship between miR-127 and Kif3b, luciferase reporter constructs carrying a 3′-UTR sequence of Kif3b (Kif3b-WT) or a mutant form (Kif3b-MUT) were generated (Figure 3C). Results demonstrated that the ectopic expression of miR-127 significantly decreased the expression level of luciferase with Kif3b-WT while that with Kif3b-MUT was intact in either MIN6 cells (Figure 3D) or HEK293T cells (Supplementary Figure 1F). In order to further identify the functional correlation between miR-127 and Kif3b, the expression of Kif3b was knocked down by siRNA (Supplementary Figure 1G). Figure 3E-3F and Figure 3G demonstrated downregulation of Kif3b significantly dampened the viability of MIN6 cells as well as isolated mouse islet cells, respectively. However, no difference of insulin secretion and intracellular insulin content was observed between control and Kif3b-knocked down cells (Figure 3H, I). These findings suggested miR-127 repressed β cell proliferation via down-regulating the expression of Kif3b.

### EVs secreted by MIN6 cells can be transferred to MS1 cells

**Figure 4:**
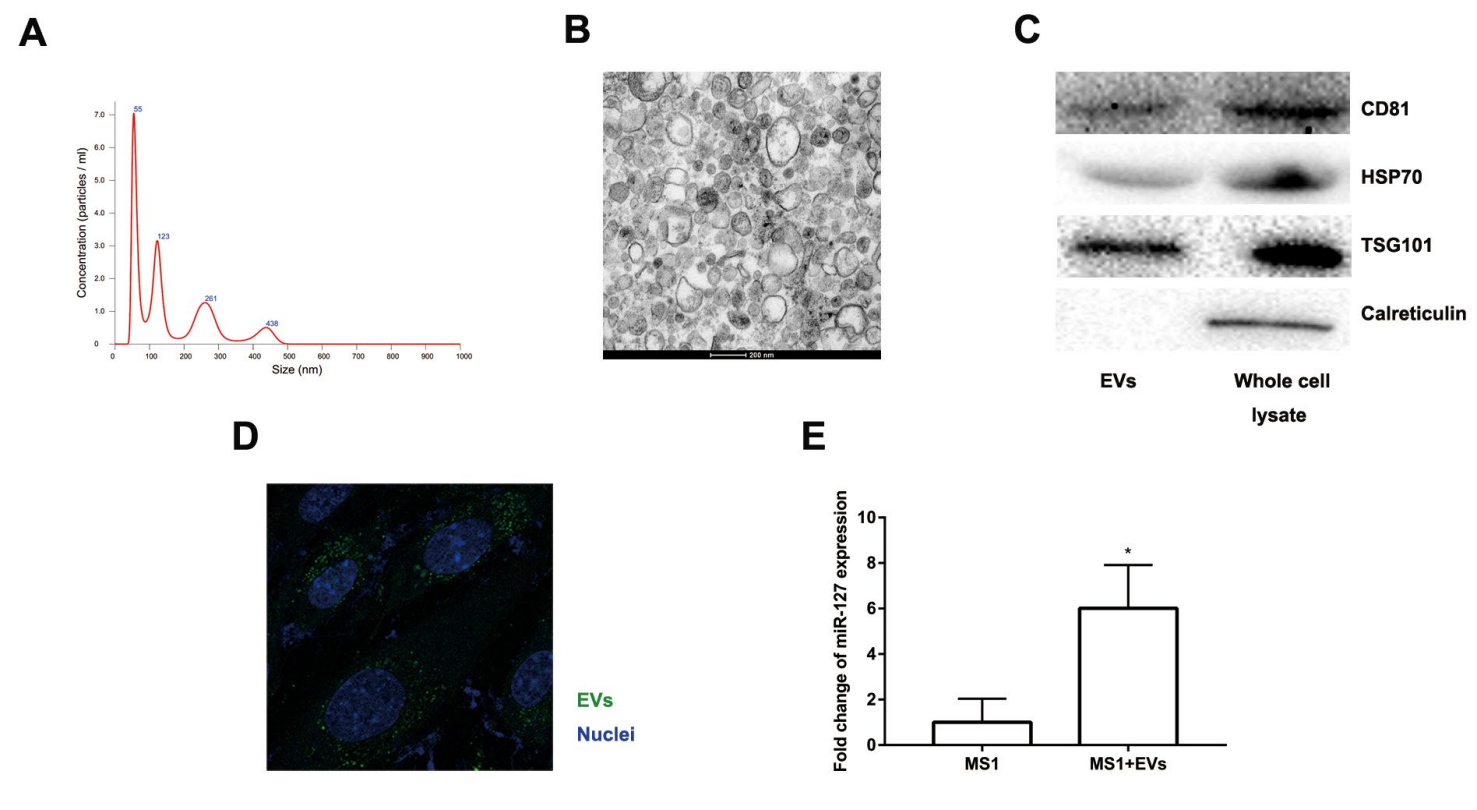
**Characteristics of EVs collected from the medium of MIN6 cells.** (**A**) Nanoparticle tracking analysis displayed the size distribution and concentration of particles isolated from MIN6 cells. (**B**) EVs were observed by transmission electron microscopy. Scale bar, 200 nm. (**C**) Western blot analysis of exosomal markers in isolated particles and whole MIN6 cell lysates. (**D**) Uptake of EVs in MS1 cells by confocal microscopy. Blue: DAPI staining; green: PKH67-labeled EVs. (**E**) The level of miR-127 in MS1 cells was measured after co-culturing with EVs for 24 h by qRT-PCR. The values are presented as the means ± SD. *p<0.05.

Accumulating evidence suggested that EVs might excrete cellular DNA or miRNA to maintain cellular homeostasis [23–25]. EVs released from β cells were involved in the cross-talk between the β cells and the endothelium and promoted vascularization [20]. Moreover, the level of miR-127 in EVs isolated from T2D patients’ plasma was significantly upregulated. The level of miR-127 was higher in islet-derived EVs than in islets [19,20]. To better clarify the function of EV-derived miR-127, we treated endothelial cells with EVs secreted by MIN6 cells in the presence of a miR-127 inhibitor or not. We isolated the EVs from the conditioned medium of MIN6 cells by centrifugation. NanoSight revealed the presence of EVs (Figure 4A). The spherical structure with bilayer membranes was observed with the transmission electron microscopy (TEM) and these particles were therefore certified as *bona fide* EVs (Figure 4B). The EV specific markers such as CD81, HSP70, and TSG101 and the ER-specific marker, calreticulin were examined in EVs or MIN6 cells lysate samples with the immunoblot assays (Figure 4C). Also, the islet endothelial cells, MS1 cells, exhibited high efficiency to uptake the EVs derived from MIN6 cells, which was detected by fluorescence microscopy (Figure 4D). Importantly, we found that the level of miR-127 was elevated in MS1 cells treated with the EVs derived from MIN6 cells (Figure 4E). Taken together, these results indicated that EVs derived from MIN6 cells could influence the islet endothelial cells *in vitro*.

### MiR-127 promoted MS1 cell migration and tube formation

**Figure 5:**
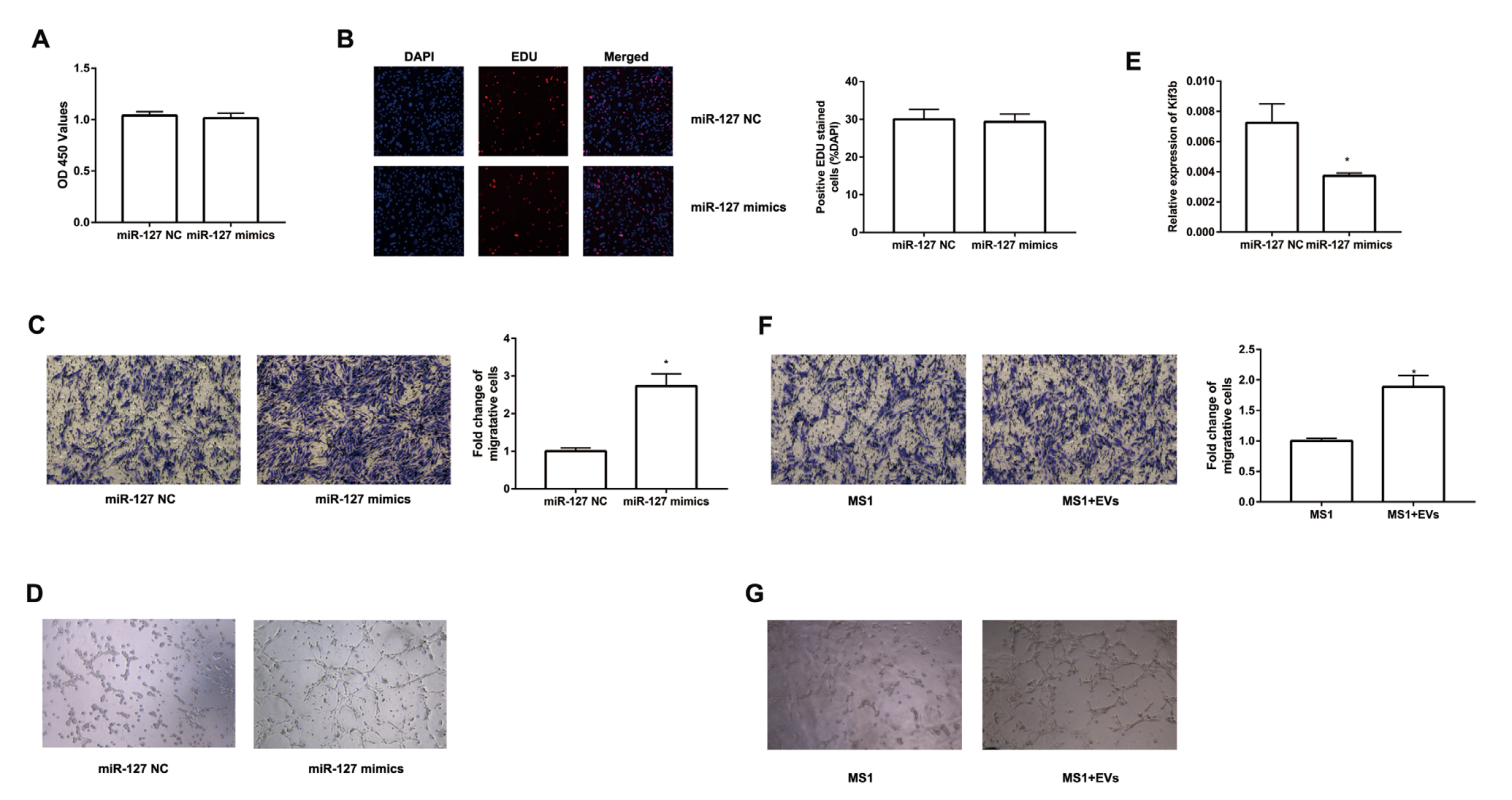
**miR-127 enhanced MS1 cells migration and tube formation.** The cell growth of transfected MS1 cells was measured by CCK8 assay (**A**) and EDU assay (**B**). (**C**) Transwell migration assays were applied to measure cell migration. (**D**) MS1 cells were seeded in pre-solidified matrigel and the indicated images were captured. (**E**) The level of Kif3b expression was measured with qRT-PCR in transfected MS1 cells. (**F**) Migration activity of MS1 cells treated with EVs was evaluated by Transwell assay. (**G**) Tube formation assay showed interference of network assembly of EVs treated MS1 cells on matrigel. The values are presented as the means ± SD. *p<0.05.

To further study the effect of EV-derived miR-127 on endothelial cells, we transfected miR-127 mimics to MS1 cells. We then evaluated the proliferation of these cells with CCK-8 and EDU assay. However, there was no significant difference between miR-127 mimics group and NC group (Figure 5A, B). Next, we examined the impact of miR-127 on the migratory capabilities of MS1 cells. As depicted in Figure 5C, migration of MS1 cells were remarkably enhanced with miR-127 mimics. Previous studies have shown increased vascularization of islets in T2D patients [26]. We revealed that MS1 cells transfected with miR-127 mimics exhibited an increase in tube formation ability (Figure 5D). Furthermore, high level of miR-127 also remarkably down-regulated the expression of Kif3b in MS1 cells (Figure 5E). To directly determine the effect of β cell-secreted EVs on MS1 cells, MS1 cells were co-cultured with the EVs. Similarly, EVs treated MS1 exhibited higher ability of cell migration (Figure 5F) and tube formation (Figure 5G). Moreover, simultaneously transfecting the miR-127 inhibitor in EVs-treated MS1 cells dampened the effect on cell migration induced by EVs (Supplementary Figure 2A, B). These data demonstrated a prominent role of miR-127 in regulating cell migration and tube formation of the endothelial cells.

## DISCUSSION

In this study, our findings demonstrated that high level of miR-127 significantly inhibited β cell proliferation and insulin secretion *in vitro*. In addition, β cell-secreted EVs containing miR-127 could promote endothelial cells migration and tube formation. Moreover, the level of miR-127 was significantly down-regulated in the islets from HFD mice. These findings suggest that a high level of miR-127 is associated with the dysfunction both in pancreatic islet β cells and endothelial cells.

The role of miR-127 in β cell function has not been reported while many studies have demonstrated either tumor-promoting or tumor-suppressive effect of miR-127 in tumor cell. For example, miR-127 reduced cell proliferation in osteosarcoma by targeting Sept8 [27]. Similarly, the tumor-suppressive effect of miR-127 were observed in giant cell tumors of bone [28]. In contrast, miR-127 promoted lung cancer development [13]. In our study, miR-127 impaired cell proliferation and insulin secretion in β cells. The differential role of miR-127 reported in various studies might result from either distinct target genes or differential effects of its target genes dependent on specific cell types.

Kinesin superfamily is a conserved class of microtubule-dependent molecular motor proteins in charge of intraflagellar transport [29]. As a member of the kinesin superfamily, Kif3b is enriched in neural tissues and involved in vesicle transport and membrane expansion [30]. Dysregulation of Kif3b has been found in many diseases including human hepatocellular carcinoma [24], acute spinal cord injury [31]and processes such as axonal regeneration [32]. In this study, we identified Kif3b as a direct target of miR-127 in the regulation of β cells. Kif3b expression knocked-down led to a reduced level of proliferation in β cells. These results are in agreement with one of the previous studies on hepatocellular carcinoma, which demonstrates that Kif3b is a potent activator of proliferation with increased expression levels in hepatocellular carcinoma tissues [22]. Similar tumor-promoting effects were also reported in seminoma [33]. However, we did not find any effect of Kif3b on insulin secretion. It is well known that numerous genes can be regulated by a single miRNA. In this report, we chose Kif3b as the target gene for the whole study. But we could not exclude the possibility that miR-127 also regulated other genes which might be involved in insulin secretion. For example, the gene, PKD1, was also predicted by Targetscan, which was reported as an essential factor in insulin secretion [34]. As PKD1 was only predicted by one algorithm tool, we did not select PKD1 as target gene for this study. It is indispensable to explore other target genes of miR-127 in our future project.

Findings from previous studies demonstrated higher levels of miR-127 in islet-derived EVs, which promoted the vascularization of islet endothelial cells [20]. Thus, it is possible that the pro-angiogenic effect of islet-derived EVs is dependent, at least in part, on miR-127. We demonstrated that elevated level of miR-127 enhanced cell migration and tube formation of the endothelial cells. This notion was further supported by the experiment performed in MS1 cells treated with the EVs derived from MIN6 cells. There was a tight relationship between islet β cells and islet vasculature. β cells might produce several angiogenic factors, such as VEGF-A to support normal islet vascularization [35]. On the other hand, islet vasculature regulates the mass and function of β cells and provides the signal for the endocrine cell differentiation [36]. When compared to healthy subjects, the islets from T2D patients displayed increased capillary density [26]. These observations raised interesting possibilities that miR-127 might be served as a therapeutic target for both β cells and endothelial cells in T2D patients.

However, there are still some limitations within this study. All experiments were done *in vitro*, which would diminish the power of our results. A β cell-specific miR-127 knockout mouse model should be used in our future studies to explore the role of miR-127 *in vivo*. To better verify the function of miR-127 in diabetes, primary islets especially human islets should also be studied in the future study.

In summary, our study characterizes the function of miR-127 in inhibiting β cell proliferation and insulin secretion. We also provide evidence to indicate a novel pro-angiogenic role of EVs released from β cells via miR-127. Finally, our findings might shed new light on therapeutic strategies to treat diabetes.

## MATERIALS AND METHODS

### Animals maintenance and islet isolation

Four weeks old female C57BL/6J mice were purchased from the Model Animal Research Center of Nanjing Medical University. One day after arrival, mice were given access to a normal fat diet (NFD, 11% calories from fat) or a high fat diet (HFD, 60% calories from fat; Dyets Inc, Bethlehem, PA, USA) for 1 week or 16 weeks. Weight and blood glucose were monitored once a week. Pancreatic islets were isolated according to previously described methods [37]. Briefly, the pancreata were digested for 12 minutes at 37°C using Collagenase P (Roche, Switzerland). Islets were then handpicked twice under a stereomicroscope. All procedures involving animals were done with approval from the Ethics Committee for the use of Experimental Animals at Nanjing Medical University.

### Cell cultures and glucose treatment

MIN6 cells (ATCC, Manassas, USA) were maintained in DMEM containing 25 mM glucose, 15% heat-inactivated fetal bovine serum, 70 µM β-mercaptoethanol. HEK 293T cells (ATCC, Manassas, USA) and MS1 cells (ATCC, Manassas, USA), a murine microcapillary endothelial cell line originated from islets of Langerhans [38], were grown in DMEM containing 25 mM glucose, 10% heat-inactivated fetal bovine serum. DMEM used for EVs isolation was depleted of EVs by overnight ultracentrifugation. Isolated islets were dissociated by trypsinization and cultured in RPMI 1640 medium with 10% heat-inactivated fetal bovine serum overnight for further usage. All cells were cultured in a humidified incubator with 5% CO_2_ at 37°C.

### Glucose treatment

To simulate high glucose condition of type 2 diabetes, MIN6 cells were exposed to DMEM consisting of 5.5, 11.1, 25, or 33.3 mM glucose for 24 h. Then, the cells and the samples of supernatant were collected for further analysis.

### Cell transfection

When the cells reached to 50%-80% confluency, the transfection was performed by using Lipofectamine 3000 (Invitrogen, Carlsbad, CA) according to the manufacturer’s instructions. A final concentration of 50 nM of miRNA mimics/inhibitor or siRNA was used for each transfection. SiRNA against Kif3b, scrambled siRNA, miR-127 normal control (NC)/mimics/inhibitor were synthesized by Genepharma (Shanghai, China). The sequences of these oligonucleotides were listed in Table 1.

**Table 1:** Primer sequence used for qRT-PCR and miR-127, siRNA related sequence.

Kif3b	F: CGGCCCATGAATGGCAAAG R: CCAATCATACACAGCGTCGAAG
Wnt7a	F: CCGTTGGAACTGCTCAGCG R: CCGCAGCGATAATCGCATAG
Fjx1	F: ACCTGACAGCCAACTTCGAC R: CGGAATACACACACCGACTG
Sept7	F: GGTGAATCTGGACTGGGAAA R: CTCCAAATCCCGGAGTATCA
GAPDH	F: GCACCGTCAAGGCTGAGAAC R: GGATCTCGCTCCTGGAAGATG
U6	F: CGCTTCGGCAGCACATATAC R: AAAATATGGAACGCTTCACGA
Beta-actin	F: GGCACCACACCTTCTACAATG
	R: GGGGTGTTGAAGGTCTCAAAC
Kif3b siRNA	Sense: CCUUUCCGCCUUGGGUAATT Antisense: AUUACCCAAGGCGGAAAGGTT
Negative control	Sense: UUCUCCGAACGUGUCACGUTT Antisense: ACGUGACACGUUCGGAGAATT
mmu-miR-127 mimics	Sense: UCGGAUCCGUCUGAGCUUGGCU Antisense: CCAAGCUCAGACGGAUCCGAUU
mmu-miR-127 inhibitor	Sense: AGCCAAGCUCAGACGGAUCCGA
Negative control inhibitor	Sense: CAGUACUUUUGUGUAGUACAA

### RNA extraction and qRT-PCR

Total RNA was extracted from the cells and the tissues using TRIzol reagent (Invitrogen, USA) and quantified by spectrophotometry (NanoDrop 2000, ThermoFisher, Waltham, MA). For isolation of RNA from vesicles, cel-miR-39 (RiboBio, Guangzhou, China) was spiked in (250 fmol) prior to total RNA extraction. The extracted RNA samples were reverse-transcribed by a PrimeScript RT Master Mix kit (Takara, Japan). QRT-PCR was performed on the StepOnePlus Real-time PCR System (Applied Biosystems, CA) with SYBR Premix Ex Taq II (Takara, Japan). For the reverse transcription reaction and quantification of miRNA, Bulge-Loop miRNA qRT-PCR Starter Kit (RiboBio, Guangzhou, China) was applied. Briefly, 2μg RNA, 5nM Bulge-Loop miRNA RT specific primers, 0.2mM dNTP, 0.04U RNase inhibitor and 0.2U reverse transcriptase were mixed for cDNA synthesis. A 10 μl reaction containing 5 μl SYBR qPCR mix, 500nM forward and reverse primers, 1 μl cDNA and ddH_2_O was used for PCR amplification. GAPDH and U6 were used as endogenous reference for mRNA and miRNA respectively. Notably, miRNA expression in vesicles was normalized to cel-miR-39 and β-actin was used as endogenous reference for mRNA in glucose treatment. The sequences of primer pairs applied in RT-PCR were listed in Table 1.

### Insulin secretion assay

To investigate the secretion of insulin ex vivo, MIN6 cells were starved in Krebs Ringer buffer (KRB containing 136 mM NaCl, 4.8 mM KCl, 2.5 mM CaCl_2_, 1.2 mM MgSO_4_, 1.2 mM KH_2_PO_4_, 5 mM NaHCO_3_, 10 mM HEPES and 0.1% BSA, pH 7.4) with 2 mM glucose for 30 minutes. The cells were then incubated in KRB containing 2 or 20 mM glucose for 1 h. After 1 h, the supernatant was collected to measure the level of insulin secreted by MIN6 cells. The cells were also collected to estimate intracellular insulin content with acid-alcohol extraction. Insulin was then assayed using a mouse-insulin ELISA kit (Mercodia, Uppsala, Sweden). For the normalization of insulin content, protein content of each well was extracted with RIPA buffer and measured by the Bradford method (Beyotime, Nantong, China).

### Tube formation

Briefly, a 48-well plate was coated with 100 μl growth factor-reduced Matrigel (BME, Trevigen) and allowed it to polymerize for 30 min before use. MS1 cells with indicated treatments were then seeded into each well. After 4h incubation at 37°C, the representative images were captured under a microscope with a 10x objective. Tube length was quantified by Image J.

### Transwell migration assay

Cell migration ability was measured by using Transwell chambers (Corning, USA). A total of 3 × 10^4^ cells suspended in 100 μl medium without serum or growth factors were seeded onto the upper wells and 400 μl medium supplemented with 10% serum was placed in the lower chamber as a chemoattractant. After incubation for 24 h, cells on the lower membrane surface were fixed with methyl alcohol and stained with 0.5% crystal violet solution. Images were taken in five random fields at 10x magnification using an inverted microscope.

### CCK-8 assay

Transfected cells (1 × 10^3^ per well) in 100 μl complete medium were seeded in 96-well plates and cultured overnight. After the overnight culture, the cells were treated with CCK-8 solutions (Beyotime, Nantong, China) and incubated at 37 °C for 2 h. The absorbance at 450 nm was measured by a microplate reader.

### Cell cycle

Forty-eight hours after transfection, the adhered cells were collected and fixed in 75% ethanol overnight at −20 °C. These cells were subsequently incubated with a mixture of 50 mg/ml PI (Sigma-Aldrich) and 25 mg/ml RNase A for 30 min in the dark. Cell cycle status was analyzed with a flow cytometer, FACS Calibur (BD Biosciences, San Jose, CA).

### Western blot

Total protein was extracted from cells and tissues using RIPA lysis buffer and then quantified with the Bicinchoninic Acid assay (Beyotime, Nantong, China). An equal amount of protein was separated by SDS-polyacrylamide gel electrophoresis (SDS–PAGE) and transferred to PVDF membranes (Millipore, Burlington, MA). After blocking with 5% skim milk for 1h, the membranes were incubated with the primary antibodies specific to the following antigens: Kif3b (Sigma, St. Louis, MO), CD81 (Abcam, Cambridge, UK), HSP70 (Abcam), TSG101 (Abcam), β-tubulin (Abcam), calreticulin (Abcam, UK) at 4°C overnight. The membranes were then incubated with horseradish peroxidase-labeled secondary antibodies at room temperature for 1 h. ECL Plus Western Blotting Substrate (Thermo Fisher, Waltham, MA) was used to visualize the immunoreactive bands. β-tubulin was used as the internal control.

### Dual-luciferase reporter assay

5×10^5^ HEK 293T or MIN6 cells were seeded in 24-well plates overnight before transfection. Then, 150 ng of wild-type or mutant Kif3b-3′ UTR dual-luciferase reporter and 50 nM miR-127 mimic or NC duplexes were co-transfected into HEK 293T cells or MIN6 cells using Lipofectamine 3000. Forty-eight hours after transfection, the cells were lysed and the activities of Firefly and Renilla luciferase were measured according to the manual of Dual-Luciferase Reporter Assay System (Promega, Madison, WI). Relative luciferase activity was normalized based on Renilla luciferase activity.

### EDU assay

Twenty-four hours after transfection, the cells were maintained in serum-free media for 24 h to allow synchronizing the cell cycle. Then, these cells were cultured for 48 h in complete medium and exposed to 50 μM EdU (RiboBio, Guangzhou, China) for the final 2 h of incubation before fixation by formalin and subsequent EdU detection following the manufacturer’s protocol. Stained cells were visualized by fluorescence microscopy (40x).

### Cell apoptosis

*In vitro* apoptosis assay was performed using Annexin V-FITC/PI staining according to the manufacturer’s protocol (Kaiji, Nanjing, China). After transfection, 5 μl Annexin V-FITC and 5 μl PI were added into 1×10^5^ cells, and incubated for 15 min at RT in the dark. Cell apoptosis was analyzed by flow cytometry.

### Extracellular vesicles (EVs) isolation

Culture medium with indicated treatment was collected for EVs isolation with sequential ultracentrifugation at 4 °C. Briefly, collected medium was centrifuged at 2,000g for 15 min and 12,000g for 30 min (Beckman, Brea, CA) to remove dead cells and cell debris. Then supernatant was filtered using a 0.22 μm filter (Millipore, Burlington, MA), followed by ultracentrifugation at 120 000 g for 2 h. The pellets were collected for both experimental treatment and storage at −80°C before use. Relative purity of the EVs were confirmed by Nanoparticle Tracking Analysis (NTA), transmission electron microscopy (TEM) and immunoblot.

### Nanoparticle Tracking Analysis (NTA)

NTA was carried out using Nanosight NS300 equipped with sCMOS camera (Malvern, UK) on samples enriched with EVs at a concentration of approximately 2×10^8^ particles/ml according to the manufacturer’s instructions. A 60 s video was recorded for further analysis by NTA software. All data was obtained at room temperature.

### Transmission electron microscopy (TEM)

EV-enriched samples were fixed with 4% paraformaldehyde and 4% glutaraldehyde in 0.1 M buffered phosphate (pH 7.4) for 30 min at 4°C. After fixation, the samples were placed on the grids and immersed in 2% phosphor tungstic acid solution (pH 7.0) for 30 s. The grids were then getting dried and the images were taken by TEM (JEM-2100 JEOL, Tokyo, Japan) at 80kV.

### EVs treatment and labelling

For EVs treatments, the cells were seeded in 12- or 96-well plates and allowed to grow overnight. On the following day, they were co-cultured with EVs at various concentrations from 0 to 200 μg exosome protein for 12-24 h. For EVs labelling experiments, purified EVs were stained with green PKH67 fluorescent dye (Sigma-Aldrich, USA) for 5 min and washed in 20 ml of PBS to get rid of the excess dyes. After centrifugation, collected EVs were incubated with MS1 cells at 37 °C for 12 h. The uptake of PKH67-labeled EVs was observed with confocal microscopy (CarlZeiss LSM710, Germany).

### In silico prediction target genes

For the prediction of the targets of miRNA, two algorithm tools, TargetScan (http://www.targetscan.org) and miRDB (http://www.mirdb.org/miRDB/) were applied. We selected the overlapping genes predicted by two algorithm systems as potential target genes.

### Statistical analysis

All experiments were performed at least three times, and only the representative results were shown. For quantitative tests, data were expressed as mean ± standard deviation (mean ± SD) and analyzed with SPSS 21.0 software (Chicago, IL). Student’s t-test was used to analyze differences between two experimental groups and p < 0.05 was considered statistically significant.

## SUPPLEMENTARY MATERIAL

Supplementary File
