# Supplementary material for: MicroRNA-127 inhibits cell proliferation via targeting Kif3b in pancreatic β cells
Source: Aging (Albany NY). 2019 Mar 1;11(5):1342–55. doi: 10.18632/aging.101835 (PMC6428088; doi:10.18632/aging.101835)
Supplement: Supplementary File [file aging-11-101835-s001.pdf]

SUPPLEMENTARY MATERIAL

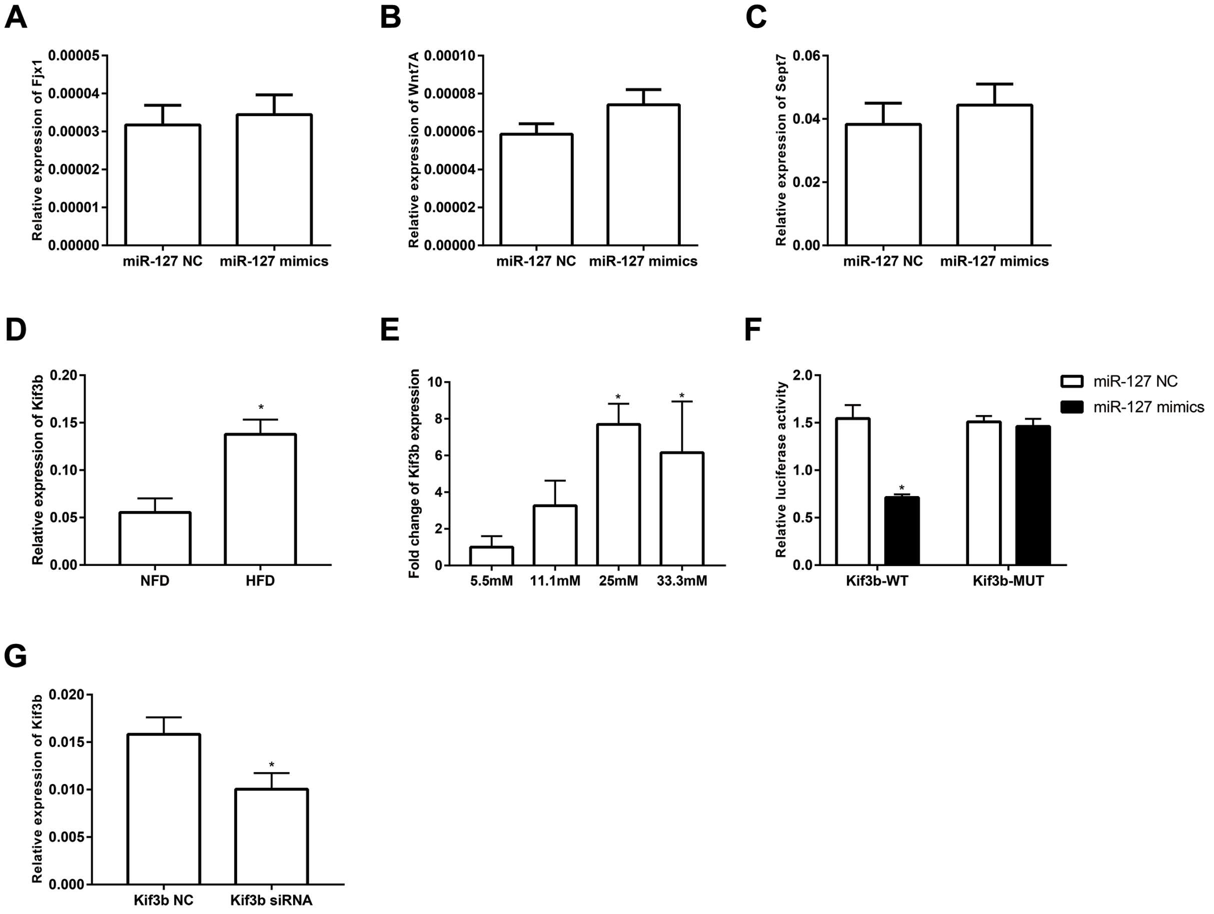

Supplementary Figure 1. miR-127 did not affect expression of other predicted target genes. The expression level of the candidate genes: Fjx1 (**A**), Wnt7a (**B**), Sept7 (**C**) in miR-127 NC or miR-127 mimics transfected MIN6 cells. Expression of Kif3b in 20-week old HFD/ NFD islets (**D**) and in MIN6 cells cultured with the indicated concentrations of glucose (**E**) was measured by qRT-PCR. (**F**) Luciferase assay of the reporter plasmids harboring the intact and mutant Kif3b-3'UTR in HEK293T cells. (**G**) Efficiency of siRNA-mediated Kif3b knockdown was analyzed by qRT-PCR.

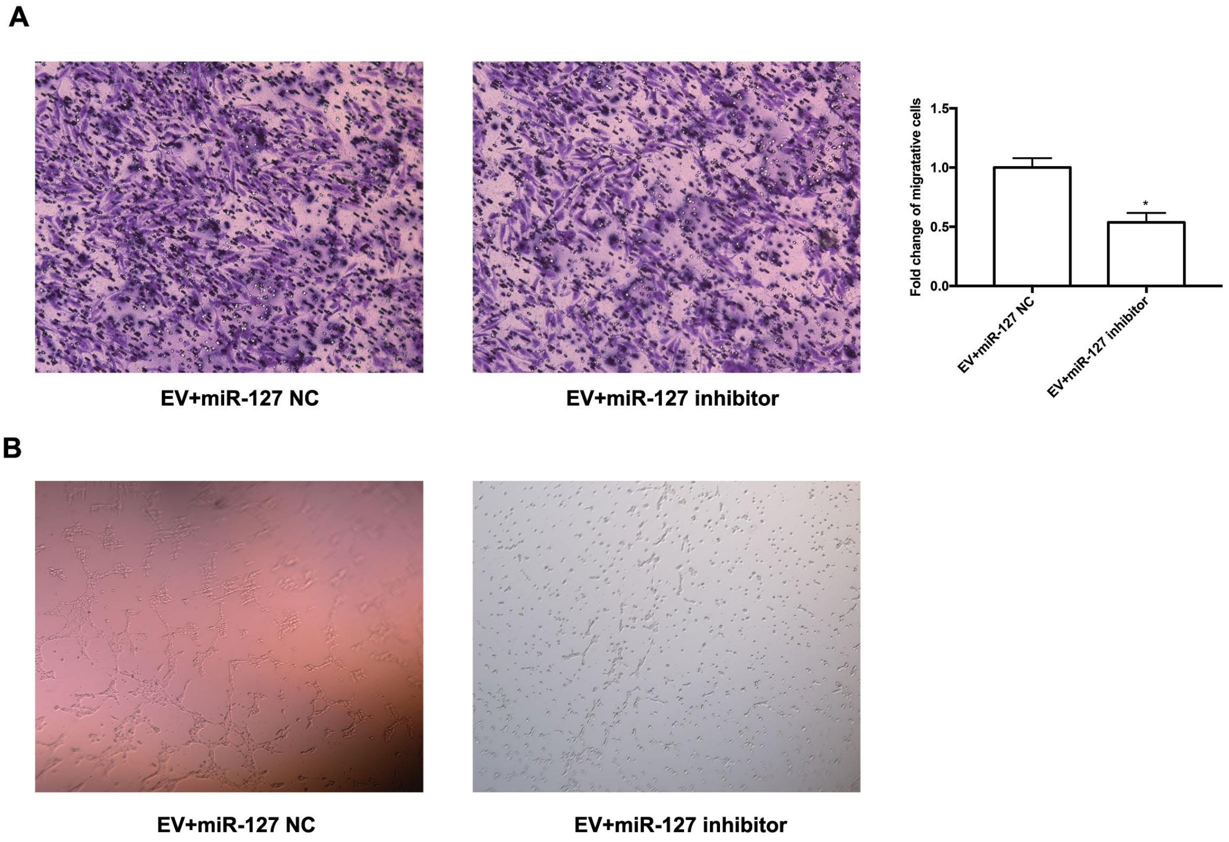

Supplementary Figure 2. miR-127 inhibitor reversed the enhanced MS1 cells migration and tube formation by EVs secreted from MIN6 cells. (**A**) Transwell migration assays and (**B**) tube formation assays were applied to explore cell migration and *in vitro* angiogenesis, respectively.
